# Lung stress, strain, and energy load: engineering concepts to understand the mechanism of ventilator-induced lung injury (VILI)

**DOI:** 10.1186/s40635-016-0090-5

**Published:** 2016-06-18

**Authors:** Gary F. Nieman, Joshua Satalin, Penny Andrews, Nader M. Habashi, Louis A. Gatto

**Affiliations:** Department of Surgery, SUNY Upstate Medical University, 750 E. Adams Street, Syracuse, NY 13210 USA; Department of Trauma Critical Care Medicine, R Adams Cowley Shock Trauma Center, Baltimore, MD USA; Biology Department, SUNY Cortland, Cortland, NY USA

## Abstract

It was recently shown that acute respiratory distress syndrome (ARDS) mortality has not been reduced in over 15 years and remains ~40 %, even with protective low tidal volume (LVt) ventilation. Thus, there is a critical need to develop novel ventilation strategies that will protect the lung and reduce ARDS mortality. Protti et al. have begun to analyze the impact of mechanical ventilation on lung tissue using engineering methods in normal pigs ventilated for 54 h. They used these methods to assess the impact of a mechanical breath on dynamic and static global lung strain and energy load. Strain is the change in lung volume in response to an applied stress (i.e., Tidal Volume-Vt). This study has yielded a number of exciting new concepts including the following: (1) Individual mechanical breath parameters (e.g., Vt or Plateau Pressure) are not directly correlated with VILI but rather any combination of parameters that subject the lung to excessive dynamic strain and energy/power load will cause VILI; (2) all strain is not equal; dynamic strain resulting in a dynamic energy load (i.e., kinetic energy) is more damaging to lung tissue than static strain and energy load (i.e., potential energy); and (3) a critical consideration is not just the size of the Vt but the size of the lung that is being ventilated by this Vt. This key concept merits attention since our current protective ventilation strategies are fixated on the priority of keeping the Vt low. If the lung is fully inflated, a large Vt is not necessarily injurious. In conclusion, using engineering concepts to analyze the impact of the mechanical breath on the lung is a novel new approach to investigate VILI mechanisms and to help design the optimally protective breath. Data generated using these methods have challenged some of the current dogma surrounding the mechanisms of VILI and of the components in the mechanical breath necessary for lung protection.

“Life leaps like a geyser for those who drill through the rock of inertia”

Alexis Carrel

## Background

The mechanisms by which mechanical ventilation exacerbates the lung damage associated with the acute respiratory distress syndrome (ARDS) have been extensively studied ever since Webb and Tierney demonstrated that high inflation pressure caused edema and that application of positive end expiratory pressure (PEEP) could protect the lung and prevent edema formation [1]. It is now known, in general terms, that the mechanisms of this ventilator-induced lung injury (VILI) are alveolar overdistension (volutrauma), alveolar instability leading to alveolar collapse and reopening with each breath (atelectrauma), and the secondary inflammation caused by these mechanical injuries which is known as biotrauma [2]. While dozens of ventilator settings were designed to block these three VILI mechanisms, with some studies demonstrating reduced mortality [3, 4], however, a recent review showed that ARDS mortality remains at ~40 % [5]. Although ARDS mortality is complex and the result of VILI is direct, clearly there is a pressing need to “drill through the rock of inertia” and entertain novel approaches to reduce ARDS mortality.

Dr. Burkhard Lachmann proposed in 1992 that the optimal lung protective strategy would be to “open the lung and keep it open” [6]. He hypothesized that heterogeneous lung inflation, which is a hallmark of ARDS pathology, plays a major role in driving mechanical ventilation-induced progressive acute lung injury. The corollary to this hypothesis is that keeping the lung open would result in a homogeneously ventilated lung, minimizing VILI and reducing ARDS mortality. If the approach of opening the ARDS lung and keeping it open can significantly reduce injury, then protective mechanical ventilation should be applied early in patients at a high risk of developing ARDS, in an attempt to “never let the lung collapse” and significantly reduce ARDS incidence [7].

## Main Text

Dr. Gattinoni’s group applies engineering concepts as a novel approach to analyze the pathologic impact of mechanical ventilation on normal pulmonary tissue and to determine what adjustments in the mechanical breath can block progressive acute lung injury and thus reduce ARDS incidence [8, 9]. In their most recent paper by Dr. Protti, their goal was to identify the volumetric threshold for VILI and determine if PEEP was directly or indirectly protective in normal pigs [9]. Unlike many experiments in which the role of tidal volume (Vt), plateau pressure (*P*_plat_), and PEEP were correlated with VILI, Dr. Protti analyzed the mechanism of VILI under two main categories with two respective subcategories: (1) *global strain* (dynamic and static strain) and (2) *energy load* (dynamic and static) within the volumetric constraints of the lung, which is the *inspiratory capacity*. Strain is the response to an applied stress, which in the case of the lung are Vt and PEEP; thus, global strain is the result of Vt + PEEP volume. Dynamic strain is the amount the volume change caused by the Vt over the FRC, and static strain is the volume change from PEEP over the FRC. Global energy load is a combination of the static component due to PEEP (conceptually equivalent to potential energy) and the dynamic cyclic component due to the driving pressure defined as Vt above PEEP (conceptually equivalent to kinetic energy).

## Discussion

Pigs were ventilated by Protti et al. for 54 h with multiple combinations of dynamic and static strain and dynamic and static energy load in three groups that were either below, within, or above the normal range of inspiratory capacity. VILI was defined as either death or development of pulmonary edema. Injury could manifest rapidly as *stress at rupture* with massive pneumothorax, or more slowly as progressive edema. In the *Below* group, there were no deaths or edema and only a slight reduction in oxygenation and elastance, so VILI did not occur in this group with any ventilation strategy. In the *Within* group ventilated with a high dynamic strain, there was an increase in edema, associated with a sharp deterioration of gas exchange and impaired lung mechanics, and 54 % of the animals died. Sixty-six percent of the pigs in the *Above* group died of stress rupture with massive pneumothorax, without pulmonary edema, and PEEP increased mortality at the same Vt.

The results from this study raise some very interesting and exciting concepts dealing with how the parameters of the mechanical breath (i.e., airway pressures, volumes, flows, and rates) injure or protect lung tissue and may help to elucidate the mechanisms of VILI. In addition, the impact of these breath parameters are analyzed as the *stress* delivered to the lung, and the response of the lung to this applied stress is measured as a *strain*. The study shows that if the stress is sufficient to exceed the inspiratory capacity of the lung, then stress failure occurs (i.e., pneumothorax); thus, in ventilation within this anatomical threshold of the lung, the main mechanism of VILI is dynamic strain. The unifying explanation is that the trigger for VILI is an excessive energy/power load, which encompasses all of the mechanical breath parameters.

Important concepts drawn from the Protti et al. study, as they apply to our understanding of VILI mechanism, would include:

1. Individual mechanical breath parameters (e.g., Vt or *P*_plat_) are not directly correlated with VILI, but rather, any combination of parameters that subject the lung to excessive dynamic strain and energy/power load will cause VILI.
2. Changing one single breath parameter, such as lowering Vt or *P*_plat_, will not protect the lung unless there is a concomitant reduction in dynamic strain and energy/power load.
3. If the inspiratory capacity is not exceeded, stress failure does not occur. Within this inspiratory capacity, dynamic strain is most injurious, as compared with static strain. Above the inspiratory capacity, the lung is exposed to stress failure.
4. All strain is not equal; dynamic strain resulting in a dynamic energy load (i.e., kinetic energy) is more damaging to lung tissue than static strain and energy load (i.e., potential energy).
5. It is clear that the independent variable for VILI is dynamic strain (Vt), while PEEP is only “protective” if it is associated with a lower Vt. PEEP can be harmful in ventilation above the inspiratory capacity.
6. For a given stress, lung strain may be completely different depending on the size of the lung. Thus, a given stress, which does not injure the normal lung, may cause severe injury in ARDS where there is a significant loss of lung volume due to atelectasis (i.e., “baby lung”).
7. A critical consideration is *not just the size of the Vt but the size of the lung that is being ventilated by this Vt.* This key concept merits attention since our current protective ventilation strategies are fixated on the priority of keeping the Vt low. Yes, low Vt is important if there is significant lung collapse (i.e., increase strain for any given stress); however, a large Vt will not cause tissue injury if the lung is fully recruited, since there are 380,000,000 inflated alveoli to share the strain. The impact of any given Vt can be assessed by changes in driving pressure calculated as the Vt/lung compliance.
8. In ARDS, we must also consider the “inhomogeneity” factor. Unevenly distributed volumes and pressures in the lung are known as stress concentrators because they induce focal stress/strain which can amount to approximately double that computed for the whole lung. Thus, in the heterogeneous lung of an ARDS patient, it is impossible to forecast whether a given “low” Vt will be safe. Further, the repetitive transition between static and dynamic strain is an important energy load factor given its potential to aggravate lung tissue stress/injury, especially in a heterogeneous structure.
9. A given airway pressure may result in widely different transpulmonary pressure (*P*_tp_), which is the stress applied to the lung, depending on the relationship between lung and chest wall elastance.
10. Driving pressure has been advocated a variable most related to VILI. However, driving pressure should be considered in relation to chest wall elastance, lung size, homogeneity, and gas flow rate. Depending on how variables may combine, the same driving pressure may be lethal or innocent.

Our commentary will focus on how these new concepts on the mechanisms of VILI-induced lung damage can help to interpret the results of other studies by using an ARDS clinical trial and a clinically applicable animal ARDS model as examples.

Recently, Amato et al. did a retrospective analysis on the data from the ARDSnet low tidal volume study [10]. These original studies set the stage for the current standard of care protective ventilation strategy of 6 cc/kg Vt and keep *P*_plat_ below 30 cm H_2_0 and PEEP set on a sliding scale based on changes in oxygenation. Surprisingly, Amato’s study showed that Vt, *P*_plat_, and PEEP had no correlation with patient mortality. What did correlate with mortality was the *driving pressure*—expressed as Vt/lung compliance. Thus, the individual parameters of the mechanical breath cannot be used in a one-size-fits-all protective ventilation strategy but rather must be personalized and adaptively adjusted based on changes in patient’s lung physiology. In the case of driving pressure, the change physiology is lung compliance. Thus, if the ventilator is set to minimize dynamic strain and energy/power load, which can be assessed by the driving pressure, VILI will be minimized and outcome improved.

**Fig. 1:**
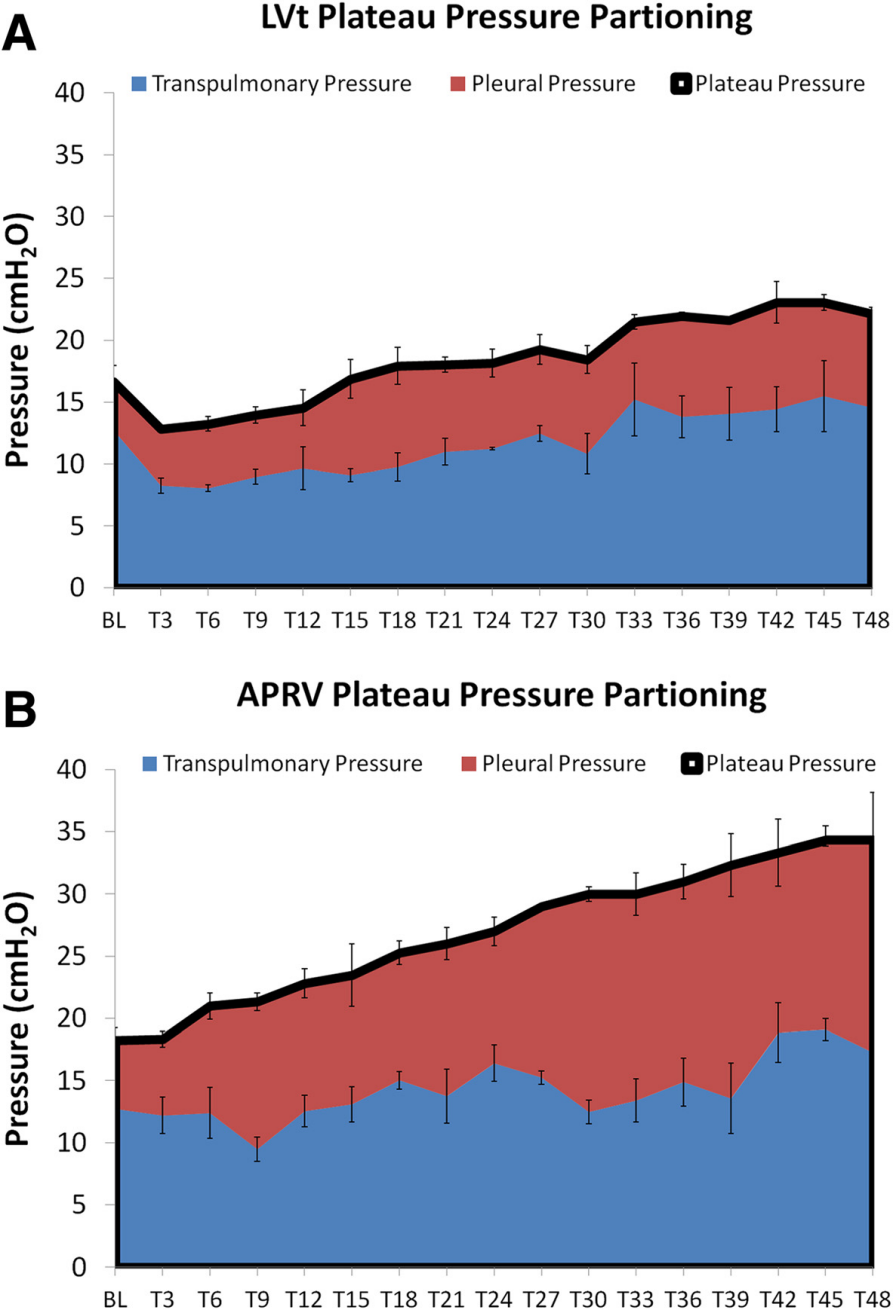
The plateau pressure (*black line at top of the red area curve*) in LVt (**a**) is significantly lower than that of APRV (**b**) yet the transpulmonary pressures (*blue*) are statistically similar between the groups. This demonstrates that the increases in plateau pressure in APRV reflect and increase in pleural pressure (*red*)

Kollisch-Singule et al. showed that preemptive application of airway pressure release ventilation (APRV) in a high-fidelity, clinically applicable, porcine ARDS model was lung protective despite higher plateau pressure and tidal volumes, as compared to a low Vt (LVt) ventilation strategy [11]. So, using the concepts of VILI mechanism in the Protti paper [9], how can these results be explained? First, the transpulmonary pressure (*P*_tp_), and thus the lung stress, was similar between the groups despite the fact that *P*_plat_ and Vt were higher in the APRV group (Fig. 1). Therefore, the increased *P*_plat_ in the APRV group was transmitted as pleural pressure and applied to the chest wall without an increase in dynamic strain or energy/power load to the lung.

## Conclusions

In conclusion, analyzing the impact of the mechanical breath on the lung using engineering concepts is an exciting new approach to investigate the mechanisms of VILI and to help design the optimally protective breath. This optimal breath must be personalized with the ability to be continually adjusted by changes in lung pathophysiology, to adaptively keep the lung open and stable. The key to the optimally protective breath is to minimize dynamic strain and energy/power load. As long as these components are held in check, the lung will be protected from VILI. Thus, high tidal volume or plateau pressures are safe as long as dynamic strain and energy/power load are maintained in the safe range [10, 11]. The optimally protective breath must be matched to the patient’s complex pathology taking into account the compliance of the chest wall and the possible presence of inhomogeneity leading to stress concentrators. Improved understanding of how to match the physics of the mechanical breath with the pathophysiology of the lung will ultimately result in the optimally protective mechanical breath.
